# Factors Influencing Educators’ Perspectives on Accepting Extended Reality in Health Care Education: Qualitative Study

**DOI:** 10.2196/65042

**Published:** 2025-05-01

**Authors:** Zuheir Khlaif, Nisreen Salama, Bilal Hamamra, Allam Mousa

**Affiliations:** 1 Faculty of Humanities and Educational Sciences An-Najah National University Nablus Occupied Palestinian Territory; 2 Faculty of Nursing Arab American University Jenin Occupied Palestinian Territory; 3 Faculty of Engineering An Najah National University Nablus Occupied Palestinian Territory

**Keywords:** extended reality (XR), health care education, educational technology, Sustainable Development Goals (SDGs), Palestine

## Abstract

**Background:**

Palestinian higher education institutions face limitations in providing interactive practical training for medical education. Extended reality (XR), which encompasses virtual reality and augmented reality, is increasingly recognized for addressing these challenges by offering immersive learning experiences.

**Objective:**

This study investigates the factors influencing the acceptance and adoption of XR in health care education within Palestinian universities, exploring its potential to transform traditional teaching methods.

**Methods:**

A qualitative approach was used in this study to collect data through semistructured interviews and artifacts from the participants. The participants of the study were 25 faculty members from 2 large Palestinian universities who teach in the field of medical sciences.

**Results:**

Three primary categories—external, internal, and design-related factors—emerged as pivotal in influencing XR adoption. Professional development, technical support, and infrastructure were key external enablers. Internally, prior experience with digital tools and positive attitudes had a significant impact on the adoption of XR. Design factors, including ease of use and interactivity, played a crucial role but also posed challenges for less tech-savvy educators. Despite barriers such as cost and technical issues, XR demonstrated notable benefits, including enhanced learning outcomes, improved knowledge retention, and the ability to simulate complex medical scenarios.

**Conclusions:**

XR technologies offer transformative potential for health care education in Palestine. By addressing challenges and leveraging XR’s strengths, educational institutions can foster innovation and improve student engagement and skill acquisition. The study contributes to the theoretical understanding of technology acceptance in education by identifying the interplay of external, internal, and design factors. Practically, it emphasizes strategic investments in infrastructure, professional training, and institutional policies to optimize XR integration.

## Introduction

### Background

**Figure 1:**
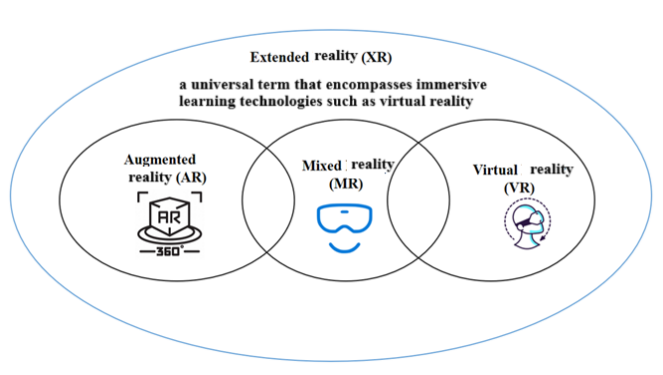
Illustration showing how extended reality (XR) encompasses augmented reality (AR), mixed reality (MR), and virtual reality (VR), as developed by the authors.

The integration of emerging technologies such as artificial intelligence and XR has significantly enhanced learners’ engagement and interaction with educational environments [1]. XR, an umbrella term encompassing virtual reality (VR), augmented reality (AR), and mixed reality (MR), facilitates human-machine interaction through computer-generated content [2]. VR provides a fully immersive digital environment, AR overlays virtual objects onto real-world settings, and MR blends physical and digital elements into a seamless interactive experience [2]. By incorporating visual, auditory, and interactive elements, XR enhances real-world learning by immersing students in digital environments that facilitate deeper engagement with educational content [3]. As shown in Figure 1, XR encompasses AR, MR, and VR, creating a spectrum of immersive experiences. Figure 1, developed by the authors, shows how XR encompasses AR, MR, and VR.

XR technologies are transforming medical education, particularly in resource-constrained settings, by providing cost-effective, risk-free learning environments. Head-mounted devices enable students to practice medical skills repeatedly without compromising patient safety. A review of 27 studies found XR-based training to be highly effective in surgery and anatomy, although more large-scale research is needed to fully assess its impact [4]. While some studies, such as Behmadi et al [5], found no statistically significant improvements in knowledge retention when comparing XR-based learning with traditional methods, they reported enhanced student engagement and satisfaction. This suggests that XR should be integrated alongside conventional teaching methods to cater to diverse learning styles and develop critical decision-making skills. Additionally, XR reduces dependence on costly medical equipment and provides students with virtual laboratories to conduct experiments, mitigating financial and logistical barriers [3,6]. By enabling hands-on practice in safe, controlled environments, XR improves learning accessibility and instructional quality [6].

In Palestinian higher education, XR adoption remains in its early stages, particularly in health care disciplines such as medicine and nursing. Its integration is closely tied to broader digital transformation policies, which aim to modernize educational practices and align with global trends in technology-enhanced learning. However, the transition faces barriers such as limited resources, inadequate infrastructure, and the need for faculty capacity-building. Despite these challenges, Palestinian universities are increasingly prioritizing XR within their strategic frameworks, reflecting a commitment to leveraging emerging technologies to enhance learning outcomes.

This study contributes to the growing research on XR adoption by examining Palestinian higher education institutions, a unique and underrepresented context. Unlike studies from technologically advanced regions, where XR adoption is often supported by substantial funding and infrastructure, this research highlights the policy-related, infrastructural, and faculty-specific challenges of implementing XR in resource-limited settings. While previous studies in Latin America and Southeast Asia have explored XR’s role in medical education, they have often overlooked institutional policies, faculty readiness, and regional constraints. By addressing these factors, this study provides a comparative perspective on XR adoption in Palestinian universities and offers global insights into key determinants for sustainable technology integration in medical education.

### Literature Review

#### The Unified Theory of Acceptance and Use of Technology Model in XR Adoption for Health Care Education

##### Overview

VR and AR are highly adaptable technologies that use various systems, setups, and content types, ranging from immersive and dynamic to nonimmersive and static environments. These technologies are characterized by 3 key elements: immersion, presence, and interaction [7,8]. Immersion depends on the technological medium, such as head-mounted displays, concave or 3D projections, or interactive videos where users engage as protagonists. Presence and interaction, by contrast, relate to an individual’s perception of connectedness within the virtual environment and their ability to act upon it and receive feedback [7,8]. These elements are crucial in defining the effectiveness and adoption of XR technologies, particularly in educational contexts such as health care training.

The Unified Theory of Acceptance and Use of Technology (UTAUT), developed by Venkatesh et al [9], serves as the theoretical framework for examining the adoption of XR in health care education. Over time, various theories and models have been proposed to understand the factors influencing the acceptance of new technologies. One of the foundational models in this field is the Technology Acceptance Model (TAM), developed by Davis [10] and based on the Theory of Reasoned Action. The TAM has been widely used to investigate the adoption of emerging technologies, including XR in surgical training, artificial intelligence adoption, and mobile learning [11-13]. However, the TAM has been critiqued for its limited predictive accuracy, as it fails to account for technology acceptance in nearly 40% of cases [14].

To address these limitations, Venkatesh et al [9] developed the UTAUT, which integrates multiple previous models, including the Theory of Reasoned Action, to provide a more comprehensive framework for understanding technology adoption. The UTAUT identifies 4 key constructs that shape an individual’s intention to use, as well as their actual usage behavior of a new technology: Performance Expectancy, Effort Expectancy, Social Influence, and Facilitating Conditions.

##### Performance Expectancy

In the context of XR adoption in health care education, performance expectancy refers to the extent to which faculty members perceive XR as beneficial for both educators and students. XR facilitates immersive learning, enhances knowledge retention, and allows for realistic simulations of medical procedures without risks to patients. Faculty members recognize its potential to improve teaching effectiveness, help achieve learning objectives, and develop students’ practical skills in a safe environment.

##### Effort Expectancy

Effort expectancy concerns the perceived ease of use of XR technology in teaching and learning environments. Faculty members’ willingness to integrate XR depends on how intuitive and user-friendly the technology is. Instructors with prior experience using digital tools often find XR more accessible, while others may require training and technical support to overcome usability challenges.

##### Social Influence

The role of social influence in XR adoption is significant, as faculty members’ decisions are shaped by the expectations of colleagues, mentors, and students. In this study, social influence extends beyond professional networks to include university policies, peer recommendations, and online technology communities that encourage XR integration.

##### Facilitating Conditions

The successful adoption of XR technology in health care education depends heavily on facilitating conditions, including institutional support, infrastructure, and technical assistance. Universities must provide adequate resources, professional development programs, and robust digital infrastructure to support faculty members’ continued use of XR. Without sufficient technical support and access to XR-compatible hardware, faculty adoption may be hindered.

#### Virtual Reality in Health Care

VR technology has revolutionized health care education by offering immersive, interactive learning experiences. It creates realistic simulations that allow students to practice procedures and decision-making without risks [15]. For instance, the Stanford Virtual Heart Project uses VR to help students understand cardiac anatomy through 3D models. Furthermore, VR allows for detailed exploration of the human body, aiding in the comprehension of anatomical structures. The HoloAnatomy program at Case Western Reserve University uses Microsoft HoloLens for holographic dissections, enhancing anatomy learning and critical thinking [15].

VR provides simulation-based training for medical procedures, enhancing skills and confidence before actual patient care. Apps such as Surgical Theater aid in understanding and practicing surgical procedures [16]. VR also enables collaborative learning through merged MR and mixed XR platforms, enhancing teamwork and communication skills by combining the virtual and physical worlds [15]. VR makes learning more engaging and accessible by bringing medical simulations to remote or underresourced areas, ensuring that high-quality medical education is available to a broader audience [17].

#### XR Adoption in Education

Researchers have reported that XR is the future of learning and teaching in higher education institutions worldwide [6]. XR promotes teamwork through collaboration in VR environments [6]. Moreover, using XR in education transforms the learning process from traditional methods to active learning, where learners engage directly in learning activities [18]. In addition, XR enhances personalized learning by creating a customized learning environment tailored to the learner’s needs and abilities [19]. Educators can design digital content to simulate immersive and interactive environments, making XR particularly suitable for teaching high-risk procedures, using expensive equipment, and conducting practical training activities that require additional resources, such as those in medicine or medical sciences [20].

Other studies have confirmed that using XR in learning fosters creativity, innovation, and design among learners [21,22]. Aguayo and Eames [23] reported that virtual agents in XR facilitated language learning and teaching by making complex knowledge more accessible. Moreover, these technologies offer immersive and interactive simulations that replicate complex real-world scenarios [24]. Consequently, using XR in education enhances the real-world experience by incorporating sounds, videos, and graphics into the learning environment, enabling learners to interact more effectively [3].

#### Benefits and Challenges

VR and AR offer numerous benefits over conventional therapy, including cost reduction, fewer hospital visits for immobile patients, user-friendly experiences, and improved patient safety. They also facilitate data collection in research settings and reduce surgical errors in training [25,26]. However, many studies indicate that determining the benefits of VR and AR in health care is challenging due to their small sample sizes, heterogeneous nature, and lack of proper controls. Pain relief mechanisms remain debated, and implementation is technically complex and expensive, with lower acceptance among older adults [7,27].

One of the main advantages of using XR is its ability to provide practical training without the need for expensive physical equipment [28]. For example, medical students can practice surgical procedures in a virtual environment, reducing the need for costly medical equipment and minimizing the risks associated with real-life practice on patients [29]. The implementation of virtual laboratories through XR not only makes education more accessible but also enhances the learning experience by offering hands-on practice in a safe and controlled environment [6].

#### Factors That Influence the Continued Intention to Use XR in Health Care Sciences

A previous study explored the potential of VR and AR technologies, particularly XR, in enhancing patient care, medical education, and presurgical planning [30]. It highlights the potential of integrating XR into medical education to provide immersive, interactive experiences.

Burian et al [31] highlighted that XR technologies provide more effective training compared with traditional methods, particularly for novices. Chuah [32] conducted a study involving 45 relevant studies to assess and analyze user acceptance of XR technology from multiple theories, disciplines, and perspectives. Wearable XR technology is influenced by various factors, including cost, technical and performance issues, hardware size, sensory inputs, content quality, cognitive impacts, user satisfaction, attitudes, intentions to visit tourism sites, expectation confirmation, personality traits, knowledge transfer, support needs, presence, boundary considerations, consumer characteristics, spatial awareness, control, participation, effectiveness, familiarity, innovativeness, value perceptions, decision comfort, spatial understanding, cognitive load, virtual embodiment perception, sense of presence, health and privacy concerns, and psychological and physical risks. Curran et al [33] stated that XR technologies offer portability, standardization, replicability, accessibility, and the ability to function without heavy manikin parts. They can be widely distributed without the need for a live instructor, thereby increasing learner engagement and enhancing spatial representation.

Kluge et al [34] noted that despite limited experience with XR technology, staff and students at the University of Newcastle view it as a standard tool for teaching. They aim to develop a sustainable implementation framework within 5 years. VR, AR, and MR technologies are disrupting medical education by offering immersive experiences and alleviating traditional learning constraints. XR technologies, particularly in emergency medicine, enable remote clinical skill development, even amidst the challenges posed by COVID-19 [33]. Li and Keskitalo [35] emphasized that XR technology is commonly used in health care education for safe treatments, communication, and decision-making. It supports 5 cognitive-processing dimensions: remembering, understanding, applying, analyzing, and evaluating. AR, VR, and MR can positively impact medical education. To effectively implement XR, it is important to consider existing resources and Bloom’s taxonomy, and select the most suitable technology. Optimizing and expanding XR utilization are crucial for promoting deeper learning in health care. There is a significant gap in research regarding the factors influencing the continued use of XR in medical science, emphasizing the need for further studies in this area.

## Methods

### Study Design

We used a qualitative approach to explore faculty members’ lived experiences with using XR in medical education at 2 Palestinian universities. This approach allows researchers to gain insights into the phenomena from practitioners who are using XR in teaching [36]. We gave participants the opportunity to share their experiences with using XR in their courses. The 2 universities involved are The Arab American University-Palestine and An-Najah National University. Both universities have established digital transformation centers focused on immersive technologies to enhance teaching and learning. The Arab American University-Palestine has developed a VR laboratory within its nursing and medicine departments, using cutting-edge technology to improve educational experiences. Similarly, An-Najah National University has created VR laboratories for its Departments of Dental Medicine and Medicine, along with a general XR center that serves both the university and the surrounding community. These laboratories provide training in XR technologies for faculty and students, supporting the integration of immersive tools into the curriculum.

### Participants

This study involved 25 faculty members (18 males and 7 females) from diverse disciplines within medical sciences, representing 2 universities in the West Bank of Palestine. All participants had a minimum of 1 year of experience using XR in their teaching of undergraduate medical sciences courses, ensuring their expertise and relevance to the study’s focus. Participants contributed by engaging in semistructured interviews and providing examples of their own work and that of their students. Table 1 presents the demographic characteristics of the participants, highlighting their diversity and alignment with the study’s objectives.

**Table 1:** Demographic information about the participants in the study.

Variable and category		Frequency, n
**Gender**		
	Male	18
	Female	7
**Education level**		
	Doctorate	19
	Master’s degree	6
**Teaching experience**		
	5 years or less	5
	6-10 years	12
	11 years or more	8
**Medical sciences field**		
	Nursing	7
	Human medicine	8
	Pharmacy	4
	Dentistry	6

### Recruitment of Participants and Justification of Sample Size

This study used purposive sampling, a qualitative method that selects participants based on predefined criteria relevant to the research objectives [37]. Faculty members, aligned with the study’s focus on XR adoption in medical education, were recruited through official invitations sent by the deans of the 2 Palestinian universities.

Eligible participants were required to meet several inclusion criteria: at least one year of experience using XR in teaching, an academic position within the Faculty of Medical Sciences, and a minimum of 3 years of higher education teaching experience. Their expertise was further validated through prior research or publications on XR, active involvement in curriculum development, and proficiency with XR tools, demonstrated through certifications, training, or workshops. Additionally, student evaluations and feedback on XR-based teaching were considered.

To enhance transferability, the study included faculty from the fields of nursing, human medicine, pharmacy, and dentistry, ensuring representation across disciplines with varying levels of reliance on XR-based learning. Participants ranged from early-career faculty with 5 or fewer years of teaching experience to senior faculty with over 10 years, providing a range of professional perspectives. The sample also encompassed both experienced and novice XR users, drawn from universities at different stages of XR adoption—one with an established XR laboratory and structured training programs, and the other in an early adoption phase—offering a comprehensive view of institutional challenges and implementation strategies.

The sample size of 25 faculty members was determined based on data saturation, a key principle in qualitative research. Saturation is reached when further data collection no longer generates new themes or insights [38]. In this study, saturation was achieved by the 22nd interview, as recurring patterns related to institutional barriers, faculty perceptions, and XR adoption challenges emerged. The remaining interviews were conducted to reinforce the robustness of these themes. Prior research [9,36] on technology adoption in higher education suggests that qualitative studies with 15-30 participants provide sufficient depth to capture rich, context-specific insights. Given the exploratory nature of this study, the selected sample size was deemed appropriate for gathering in-depth faculty perspectives on XR integration in medical education.

We acknowledge that the small sample size may limit the generalizability of the findings. However, this study was designed to explore educators’ lived experiences and provide deep insights into XR adoption in medical education, rather than aiming for broad generalizability. The qualitative methodology, supported by data triangulation through interviews and artifacts, enhances the trustworthiness and validity of the findings despite the limited sample size. To mitigate this limitation, participants were purposively selected from diverse medical disciplines and institutions to ensure a variety of perspectives. Rigorous thematic analysis techniques were used, validated by multiple coders with high interrater reliability. Additionally, the findings were cross-referenced with existing literature to contextualize and strengthen the conclusions.

While future studies with larger samples are necessary to further validate these findings, this study provides valuable foundational insights into XR adoption in medical education. These findings can serve as a reference for similar educational contexts, aiding institutions and educators in navigating the complexities of XR implementation.

### Study Context

The context of this study involved faculty members from the Faculty of Healthcare Sciences at 2 major universities in the West Bank of Palestine. Both universities have a clear vision and policy to integrate emerging technologies, such as XR, into medical education and research. To facilitate the adoption of XR in teaching, 4 training workshops were organized at each university to equip faculty members with the knowledge and skills necessary to understand and apply XR in their practices. The length of each training session varied depending on the topic, generally ranging from 2 to 7 days. These sessions provided teachers with the opportunity to create and develop learning objects using XR. Various topics were covered, including an introduction to VR, AR, and XR; designing lessons using objects on the platform; and creating avatars for lessons, among others.

### Research Instrument

A semistructured interview was the primary research instrument for data collection. We developed an interview protocol to guide the process. The protocol (Multimedia Appendix 1) consisted of 2 sections. The first section introduced the study to the participants and confirmed the confidentiality of their responses. Participants were informed that the interview would be recorded, provided they agreed, and that they could withdraw at any time. They were also asked to sign a consent form before the interview was recorded. The second section contained interview questions developed from a literature review, aligned with the research questions. Another data source consisted of artifacts provided by the participants during the interviews or submitted via email, illustrating how XR was used in medical education.

### Data Collection

The researchers sent invitations to the nominated participants to schedule semistructured interviews at their convenience. Participants were given the flexibility to choose the time and location of the interviews. Each interview, lasting 30-45 minutes, was conducted individually and recorded. The interview protocol began by asking participants to discuss their general experiences with technology, followed by specific inquiries about their experiences with XR, allowing them to share their stories and journeys. Follow-up questions were asked to delve deeper into their experiences, especially regarding student collaboration on projects. For example, when one participant mentioned that students learn more through exploration, a follow-up question was, “Can you provide more details about what exploring entails and the role of XR in facilitating that exploration?” The interviews were designed to capture as much detailed information as possible from the participants’ experiences. In addition to the interviews, participants provided various artifacts, including samples of student work and activities implemented using XR. These artifacts served as valuable secondary data sources.

### Trustworthiness

To ensure trustworthiness and methodological rigor, this study followed established qualitative research principles, emphasizing credibility, confirmability, dependability, and transferability.

Credibility was enhanced through the use of multiple data collection methods, including semistructured interviews, participant-submitted artifacts, and institutional policy documents. Member checking was conducted by sharing transcribed interviews and preliminary themes with participants, enabling them to verify the accuracy of interpretations and provide clarifications where necessary. Additionally, peer debriefing was used, where external researchers reviewed the coding process and emerging themes to refine definitions, minimize bias, and ensure analytical coherence.

Conformability was maintained by carefully documenting the research process and prioritizing participants’ statements to minimize researcher bias. The interview protocol was developed based on the research questions, a pilot study, and expert reviews, ensuring its relevance and alignment with the study’s objectives. Dependability was reinforced through rigorous coding procedures, with data being independently coded 3 times, achieving 92% interrater reliability. This process ensured consistency in theme identification and analysis. Transferability was supported by purposive sampling, which selected participants from diverse academic disciplines and career stages to capture a broad range of perspectives on XR adoption. Additionally, data triangulation—incorporating interviews, observational notes, and artifacts from faculty members—provided a comprehensive understanding of the factors influencing XR integration in medical education. By incorporating these robust qualitative validation strategies, the study strengthens its credibility, validity, and applicability, ensuring that the findings accurately reflect participants’ experiences while addressing potential biases in sample selection and data interpretation.

### Ethical Considerations

This research was approved by the institutional review board committee at An-Najah National University under reference Med. April.2024/18. The study adhered to strict ethical guidelines to ensure participant confidentiality, informed consent, and data protection. Before participation, all participants were provided with detailed information about the study’s objectives, procedures, and their rights as participants. Participants were explicitly informed that their participation was entirely voluntary, and they could withdraw from the study at any time without facing any consequences or needing to provide an explanation. To document consent, participants signed an informed consent form that outlined the scope of the study, the types of data to be collected, and how the data would be used solely for research purposes. The consent form also detailed the measures taken to protect their identities and ensure the confidentiality of their responses.

In terms of data protection, stringent protocols were followed to safeguard participant information. Personal data, including contact details, were securely stored on a password-protected and encrypted computer. Both physical and digital access to these data was restricted to authorized researchers only. Furthermore, any identifying information was anonymized during the data analysis process to further protect participants’ privacy. Interview data were stored in encrypted files, and backup copies were securely maintained to prevent data loss. Additionally, the researchers communicated the steps taken to comply with ethical research standards, including adherence to the principles outlined by Ngozwana [39]. These procedures ensured that participants felt confident their contributions were protected and respected throughout the research process. By incorporating these comprehensive protocols, the study’s transparency and ethical rigor were enhanced.

### Data Analysis

To address the research questions, inductive thematic analysis was used, following the 6-step methodology proposed by Braun and Clarke [38]. The research process involved conducting 10.5 hours of recorded interviews. Before data analysis, the researchers transcribed the audio recordings and shared the text files with participants for validation. Participants were given the opportunity to amend, rewrite, or supplement the content as needed. Once the files were returned, no further modifications were expected. The thematic analysis was carried out in 6 phases, as outlined by Braun and Clarke [38], and these phases guided the data analysis process in this study.

The researchers began their analysis by organizing the individual transcript files, labeling them as F1, F2, and so on up to F25. The initial phase involved familiarizing themselves with the data. The researchers, who had conducted the interviews, carefully read through each transcript while simultaneously listening to the corresponding audio files. This process of immersion allowed the researchers to take detailed notes in the margins of the transcripts, ensuring a thorough understanding of the data. In the second phase, coding, the researchers developed labels for significant features of the data that were relevant to the research questions. As they meticulously read through the transcripts line-by-line, they created a coding book to document these labels. This phase focused on identifying key concepts or ideas that were central to the research, such as how faculty members experience XR in teaching medical concepts and in transferring medical knowledge and skills to their students. Next, the researchers moved to the theme development phase, where they identified patterns related to the research questions. They grouped similar and contrasting codes into coherent themes, resulting in a structured coding book (Table 2 and Multimedia Appendix 2). In the fourth phase, reviewing themes, the researchers examined these themes across all data, using inductive analysis to refine and combine them as needed. This iterative process of theme development was essential for generating meaningful insights from the data.

The fifth phase involved defining and naming themes based on the research questions, literature review, and theoretical framework. This phase integrated both inductive and deductive approaches, which are common in qualitative data analysis. Finally, in the reporting phase, the researchers documented and presented the analysis results, supporting the findings with direct quotations from the participants’ interviews. This approach helped provide concrete evidence and context for the identified themes.

The data analysis process began with a thorough review of each transcript to familiarize the researchers with the material. During this phase, ideas and concepts were identified as units of analysis, and a coding process was developed. A coding book was created to assist with data coding. Ideas and concepts sharing similar characteristics were organized into emerging subthemes, which were then grouped under primary themes, informed by prior research findings. The research questions guided the analysis process. Table 2 illustrates the inductive thematic analysis approach used to develop the coding book for each research question.

**Table 2:** An overview of the themes and data sources (interviews and artifacts).

Major themes and subthemes			Findings from interviews		Findings from artifacts
**External Factors**					
	Professional Development	Faculty attended training sessions on XR^a^, viewed as beneficial for skill-building and knowledge sharing.		Training materials and lesson plans demonstrated faculty engagement with XR-based teaching.	
	Technical Support	Faculty emphasized the need for technical support, linking it to continued XR use.		Technical support records showed assistance provided for troubleshooting XR-related issues.	
	Infrastructure	Challenges related to limited XR devices, weak Wi-Fi, and lack of educational resources.		Limited XR-enabled classrooms and laboratories were documented in institutional reports.	
	Social Influence	Positive influence from colleagues, social media, and university policies encouraged XR adoption.		Shared lesson plans and peer-reviewed materials indicated knowledge exchange among faculty.	
**XR Features**					
	Ease of Use	Some faculty found XR easy to use, while others struggled with the complexity of designing activities.		User guides and simplified XR tools were developed to address faculty concerns about complexity.	
	Interactivity	Interactivity was valued for facilitating learning objectives and engagement.		Lesson artifacts included interactive 3D models and gamified simulations.	
	Imagination and Immersion	Imagination linked to visualization capabilities, enhancing the learning experience.		Students’ assignments showed creative applications of XR for visualization.	
**Internal Factors**					
	Previous Experience With ICTs^b^	Prior experience with ICTs contributed to smoother XR adoption.		Faculty-created resources mirrored existing ICT teaching practices, aiding XR integration.	
	Digital Competencies	Digital literacy skills were essential for the effective use of XR in teaching.		Assessment rubrics reflected the need for digital competencies in grading XR-based tasks.	
	Attitudes Toward XR	Most faculty had positive attitudes, seeing XR as engaging; a minority found it too complex.		Students’ reflections indicated excitement about XR use; some reported difficulty in self-directed learning.	
**Design Factors**					
	Design Challenges	Time constraints and lack of technical expertise made designing XR activities difficult.		Course syllabi showed attempts to integrate XR but highlighted gaps in structured activity design.	

^a^XR: extended reality.

^b^ICT: information and communication technology.

## Results

### Key Factors

Participants identified key factors based on their experiences with XR in health care education. Faculty workload and responsibility were recognized as significant factors influencing the integration of XR into teaching practices. Additionally, experience with medical technology was found to be linked to the use of XR. Data analysis revealed various influencing factors, which were grouped into 3 categories: internal factors, design factors, and external factors, which include XR features (Table 2). Each category encompasses several specific factors, as detailed in the following sections. Table 2 provides an overview of the themes and data sources.

### External Factors

#### Institutional Drivers of XR Adoption in Teaching

External factors related to higher education institution policies and readiness, such as professional development, technical support, infrastructure, social influence, and XR features, were identified as factors that could positively influence the acceptance and continued use of XR in teaching practices.

#### Professional Development

All participants confirmed their attendance at training sessions on using XR in their teaching. These sessions covered a range of topics, from recognizing the value of XR as an advanced technology to creating lessons and activities using existing platform assets. For example, one faculty member mentioned, “The training was helpful in various aspects, such as understanding the value of XR and learning how to use it in my class activities” [D1]. Additionally, some faculty members viewed the training sessions as opportunities to share their knowledge and receive feedback, enhancing their practices in medical education. One participant noted, “I shared the activities I designed from scratch to get feedback from my colleagues. It was a good chance to share experiences and learn from others” [P5].

Integrating VR into medical education, particularly in fields such as nursing, offers students the chance to learn in authentic, immersive environments and practice practical tasks through simulations. However, some participants raised concerns that VR might not substantially enhance student learning, as they believed students could already visualize real-life situations without the need for VR. This underscores the importance of professional development programs in equipping educators with the strategies needed to design VR experiences that extend beyond imagination, offering unique, hands-on, and interactive learning opportunities that traditional methods cannot replicate.

#### Technical Support

Most participants emphasized the importance of technical support in ensuring the continued use of XR, as it helps minimize technical difficulties faced by faculty members in medical sciences. Technical support encompassed a range of services, from creating platform accounts to troubleshooting issues with platform assets and student access. One faculty member stated, “Technical support is essential to continue using XR as it’s a new technology, and I had no previous experience with it” [D25]. Some faculty members associated the availability of technical support with saving time and being able to focus more on the quality of activities and assessments. However, a few reported a lack of technical support due to insufficient staffing at the XR center. Providing technical support also positively impacted students’ timely completion of assignments and tasks using XR.

#### Infrastructure

Infrastructure plays a crucial role in the use and continued adoption of XR in medical education. Participants defined infrastructure in terms of the availability of suitable VR devices, strong Wi-Fi, and educational resources. One faculty member mentioned, “I confront challenges to find assets related to Nursing relevant to my teaching topic” [D24]. A few participants cited the lack of infrastructure as a significant challenge. For instance, one faculty member said, “I have 45 students in Human Medicine, and it’s difficult to take them all to the computer lab to use the VR devices because there is only one device” [D5].

One of the novel findings of this study is its emphasis on the intersection of institutional policies, faculty readiness, and infrastructural limitations in shaping XR adoption in medical education. While faculty members acknowledged the pedagogical benefits of XR, they also highlighted significant challenges related to institutional support and funding constraints. Unlike institutions in high-income regions, where government and private sector investments facilitate the widespread adoption of XR, Palestinian universities rely primarily on limited internal budgets and external grants. Consequently, the lack of funding for VR-compatible hardware, insufficient training opportunities for faculty, and inadequate technical support staff emerged as critical barriers to adoption. This study underscores the importance of targeted policy interventions, including faculty incentives, resource-sharing initiatives, and digital transformation strategies, to address these systemic barriers and promote sustainable XR integration.

#### Social Influence

##### Positive Influence of Colleagues

When asked about the impact of colleagues on their use of XR, most participants reported a positive influence from both within and outside the university. This influence included sharing expertise, providing technical and instructional support, and exchanging lessons and learning objects on the XR platform. One participant stated, “It was challenging to design lessons using the XR platform, so I asked a colleague for help” [D20].

##### The Power of Social Media

Some participants reported being members of social media groups focused on advanced technology in engineering, such as Twitter groups. These communities helped them exchange ideas about designing VR activities and share lessons using 3D and 360-degree techniques. One participant shared a lesson about the human body on Facebook and received feedback to improve the lesson using advanced VR features. Another participant said, “I share my lessons and activities in the group and exchange ideas on using XR in teaching various topics” [D6].

#### XR Features

##### Impact of XR Features on Adoption in Medical Education

The XR features reported by the majority of participants included ease of use, imagination, interaction, and immersion, all of which could influence the use of XR in medical education.

##### Ease of Use

Most participants highlighted the importance of XR’s ease of use in lesson activities and content presentation. Some linked the simplicity of designing activities and lessons on the platform to their intention to continue using it. One faculty member stated, “I liked using XR because it was easy to design activities to show hidden parts of the human body” [D4]. However, a few participants found XR complicated and challenging for designing lesson activities, which led them to stop using it, although they continued assigning XR-related tasks to students.

##### Interaction Feature

Many participants emphasized the role of interactive features in facilitating and achieving lesson objectives. One participant said, “interactivity is important for me and my students because it enables activities that are otherwise impossible” [D11]. They also highlighted the importance of designing interaction types between students and learning activities, as well as interactions among students.

##### Imagination and Immersion

All participants confirmed the significance of imagination in medical sciences education, which could lead to greater immersion in class activities. One faculty member reported, “My students used XR to virtually perform a surgery” [D3]. Many participants linked imagination to visualization features that attract faculty members to use XR in assignments and activities.

### Internal Factors

#### Role of Digital Competencies and Experience in XR Adoption

Internal factors included previous experience with information and communication technologies and digital competencies.

#### Previous Experience With Information and Communication Technologies

The majority of respondents indicated that previous experience with information and communication technologies and mobile technology was crucial for accepting and using XR in medical education. Experience with smartphones also facilitated their use of mobile VR for course instruction and activities.

#### Digital Competencies

In this study, digital competencies refer to the knowledge, skills, and attitudes related to XR. Most interviewees reported that their digital competencies were crucial for continuing to use XR. One faculty member stated, “My experience and knowledge are important for using XR in medical education.” [D9].

### Design Factors

Design factors refer specifically to the pedagogical and instructional aspects of XR integration, particularly in creating activities, materials, and assessments in health care. This process often requires the application of instructional design principles. Most participants indicated that their primary difficulty was designing course-related activities. Many felt they lacked the technological expertise required, and some reported insufficient time due to commitments at private hospitals and clinics.

### Attitudes Toward Using XR in Medical Education

#### Positive Attitudes

Many participants expressed positive attitudes toward using XR in medical education, attributing these attitudes to features such as interaction, visualization, and immersion. Some mentioned that the simplicity of XR saved both time and effort. For instance, one faculty member noted, “My students were excited to use XR.” [D4].

#### Negative Attitudes

A minority of participants, fewer than one-third, reported obstacles that negatively influenced their attitudes toward using XR. These challenges were related to the complexity of XR usage.

### Socioeconomic Factors and XR Adoption

Socioeconomic factors significantly influence XR adoption in Palestinian higher education, particularly in infrastructure investment, faculty training, and institutional support. The high costs of XR hardware, software, and maintenance, coupled with limited government funding and restricted access to grants, present a major challenge. Many universities struggle to scale XR beyond pilot initiatives, limiting widespread faculty adoption. A key barrier is faculty training and professional development. While some institutions offer workshops, many educators lack consistent training and technical support, resulting in uneven adoption across disciplines. Comparisons with developing regions, such as Latin America and Southeast Asia, reveal similar constraints, while well-funded institutions in South Korea and Germany address these challenges through government investment, faculty incentives, and public-private partnerships.

To bridge this digital divide, Palestinian universities must increase funding, establish training programs, and explore resource-sharing models across institutions. Addressing these socioeconomic barriers will ensure sustainable XR integration, enabling faculty to effectively utilize immersive technologies in medical education. Future research should explore scalable funding models and institutional collaborations to support long-term XR adoption. In conclusion, we visualize the factors influencing faculty members’ use of XR in medical education.

**Figure 2:**
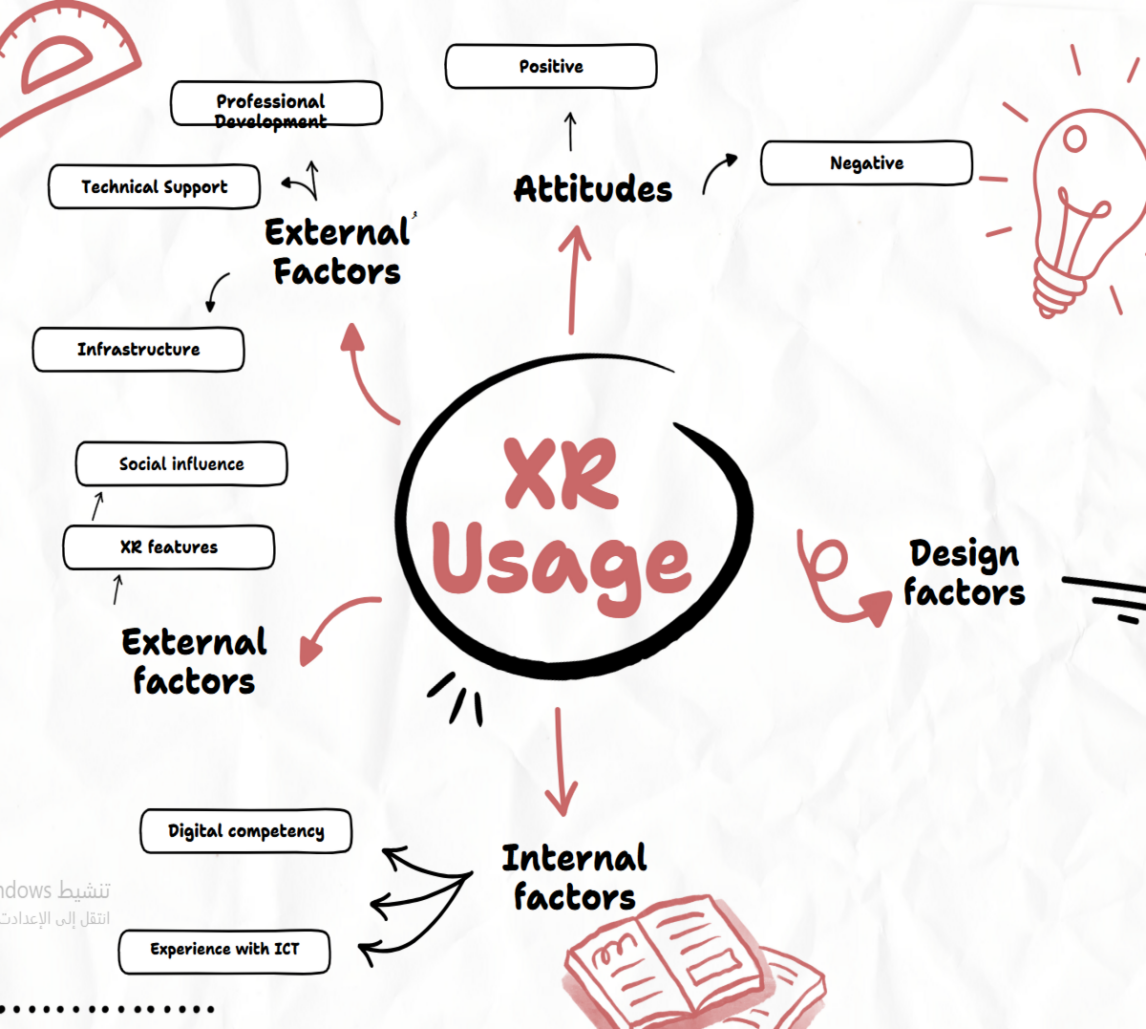
Visualization of the factors influencing faculty members to use extended reality (XR) in medical education.

Figure 2 visualizes the factors influencing faculty members’ use of XR in medical education.

## Discussion

### Principal Findings

The findings of this study highlight the complex factors influencing XR adoption in medical education, categorized into external, internal, and design-related elements. Professional development emerged as a key enabler, with faculty members who participated in XR training reporting increased confidence and capability in integrating the technology into their teaching. These sessions provided both technical knowledge and collaborative learning environments, aligning with prior research emphasizing the role of continuous professional development in technology adoption [40]. However, technical support and infrastructure remain critical challenges. While access to reliable support enhances faculty engagement with XR, inconsistent availability of assistance and limited institutional investment in technical staff hinder seamless integration. Similarly, infrastructure gaps—such as limited access to XR devices, inadequate internet connectivity, and insufficient educational resources—remain substantial barriers, especially in resource-constrained settings [41]. These findings align with studies in other developing regions, where high costs and inadequate infrastructure are primary obstacles to XR adoption [42].

Although this study applies the UTAUT model to analyze XR adoption, the findings suggest that policy and institutional support are crucial facilitating conditions not explicitly accounted for in the framework. Additionally, socioeconomic constraints, including funding limitations and digital infrastructure challenges, significantly influence adoption behaviors [43]. To extend the theoretical framework, we propose a modified model that integrates 2 additional dimensions: Pedagogical Readiness, encompassing faculty training, instructional design capabilities, and institutional encouragement for XR use, and Technical and Logistical Support, emphasizing the role of digital infrastructure, maintenance, and technical assistance. These modifications offer a more contextualized perspective on XR adoption in developing regions, reinforcing the need for localized implementation strategies [44].

The role of social influence in XR adoption extends beyond institutional policies and peer encouragement. This study found that faculty skepticism, generational differences in adoption, and student perceptions significantly influence XR use. While peer influence and institutional endorsement encourage adoption, some senior faculty members expressed skepticism about XR, fearing that it might disrupt traditional pedagogical methods rather than complement them. These concerns align with prior research on faculty resistance to emerging technologies [32]. By contrast, younger faculty members demonstrated greater openness, reflecting trends observed in broader educational technology adoption studies [30]. Additionally, students’ positive engagement with XR significantly influenced faculty willingness to integrate the technology, reinforcing prior findings that student enthusiasm can drive faculty adoption [33]. However, some educators expressed concerns that XR might encourage passive rather than active learning, highlighting the need for interactive and problem-solving–oriented XR applications to maximize its educational impact [33].

To overcome financial barriers to XR adoption in Palestinian universities, alternative and sustainable funding models are essential. While current efforts often rely on short-term external grants, more resilient approaches—such as public-private partnerships, collaborations with technology firms, and the use of open-source XR platforms—could help support long-term implementation and scalability [45]. Although the return on investment in XR may not be immediately measurable in financial terms, it can be demonstrated through improvements in student performance, engagement, and retention. These outcomes contribute to institutional sustainability by reducing dropout rates and enhancing overall learning effectiveness [41].

Lastly, design-related challenges, particularly the complexity of XR tools and time constraints for faculty, emerged as barriers to effective integration. While many faculty members appreciated the interactive and immersive capabilities of XR, others found content creation and instructional design challenging, highlighting the need for user-friendly design tools and targeted training [46]. Digital competencies were also found to be a critical factor, with faculty members possessing stronger digital skills demonstrating greater ease in XR adoption. This underscores the importance of developing digital literacy as a core competency in medical education [47].

Overall, this study emphasizes the need for a holistic approach to XR adoption, integrating technical, economic, and pedagogical strategies. In comparison to universities in Latin America and Southeast Asia, where national digital education strategies and structured funding initiatives have facilitated XR adoption, Palestinian institutions require policy-driven interventions and regional partnerships to develop scalable, sustainable funding models [48]. Addressing these economic and infrastructural constraints will be essential to ensure that XR can be effectively integrated into medical education in underresourced contexts.

### Theoretical and Practical Implications

The study on integrating XR in Palestinian health care education highlights key theoretical and practical implications. Theoretically, it advances technology acceptance models by identifying factors influencing XR adoption, including institutional policies, social influences, digital competencies, and attitudes. It also emphasizes the need for robust infrastructure and professional development to support technology integration.

Practically, effectively implementing XR in medical education requires a well-structured, phased approach to fully realize its transformative potential. The first priority for institutions should be establishing robust foundational infrastructure, including high-quality hardware and software solutions that are scalable and adaptable to evolving needs. This requires substantial initial investments, not only in technology but also in developing technical support systems to address challenges such as high costs, operational complexities, and the demands of maintaining cutting-edge solutions. Along with infrastructure development, it is essential to provide comprehensive training for educators and students, focusing on the digital literacy skills needed to use XR effectively. Clear guidelines should also be developed to ensure consistent, meaningful integration of XR into the curriculum.

Higher education institutions should also consider designing and adopting performance indicators specifically tailored to measure the success and impact of XR implementation. These indicators could include metrics such as student engagement levels, improvements in skill acquisition, and the cost-effectiveness of XR solutions. By establishing these benchmarks, institutions can monitor progress and identify areas for improvement, ensuring a data-driven approach to XR adoption.

Early adoption strategies should emphasize piloting risk-free, immersive simulations that allow educators and students to explore XR’s capabilities in a controlled environment. These pilot programs serve to demonstrate the tangible value of XR, helping to build confidence among stakeholders and secure their buy-in for broader implementation. However, institutions should be cautious of potential pitfalls. For instance, underestimating the need for ongoing technical support can lead to system failures and diminished user satisfaction. Similarly, neglecting to align XR initiatives with specific, well-defined learning outcomes can result in unfocused or ineffective use of the technology. Finally, failing to allocate adequate resources for regular maintenance and updates may jeopardize the long-term sustainability of XR programs.

### Addressing Technical and Economic Barriers to XR Adoption

Sustainable integration of XR in medical education requires a strategic focus on viable funding models, cost-effectiveness, and long-term impact. In resource-constrained settings, such as Palestinian universities, advancing XR implementation depends less on reiterating existing challenges and more on identifying innovative, context-sensitive solutions. Strategic partnerships—with private technology firms, medical institutions, and international funding bodies—can facilitate access to sponsored XR hardware, software, and training. These collaborations support the co-development of immersive learning programs and enable cost-sharing arrangements that reduce the financial burden on institutions.

Adopting open-source XR platforms also presents a promising avenue for sustainable integration. These tools offer flexibility in content creation and deployment without the high costs associated with proprietary systems, making them particularly suitable for universities with limited budgets. Beyond initial implementation, institutions must assess XR’s return on investment through educational outcomes rather than direct financial metrics. Improvements in student engagement, knowledge retention, and academic performance are strong indicators of XR’s value and can contribute to institutional sustainability by reducing dropout rates and enhancing graduate readiness.

To ensure scalability and impact, universities should adopt data-informed strategies, including cost-benefit analyses, pilot programs, and scalable deployment models. Aligning financial planning with pedagogical goals ensures that XR technologies are integrated not only as innovative teaching tools but also as sustainable investments in the future of medical education. A methodical, forward-looking approach enables institutions to transform economic limitations into opportunities for creative problem-solving and long-term growth.

### Limitations

The study acknowledges several limitations. First, the research was conducted during the initial stages of XR adoption in Palestinian higher education, which may limit the generalizability of the findings. The small sample size and the focus on specific institutions further constrain the applicability of the results to other contexts. Additionally, the high upfront costs and technical challenges associated with XR technologies may pose barriers that were not fully explored due to the limited scope of the study. Finally, the study relies on self-reported data from participants, which could introduce bias or inaccuracies in the findings.

### Future Research

Future research should focus on scaling the study to larger populations across multiple universities to provide a more comprehensive understanding of XR’s applicability and effectiveness in diverse educational contexts. Expanding research across different institutions, disciplines, and settings will offer broader insights into how XR can be integrated into various pedagogical frameworks.

Additionally, longitudinal studies are essential to track XR adoption over time. These studies would assess the long-term impact of XR on educational outcomes, skill retention, learner engagement, instructor effectiveness, and curriculum integration. Examining how faculty and students interact with XR technologies over extended periods will help identify patterns of adoption, sustained challenges, and evolving best practices. This approach will also contribute to understanding the long-term sustainability of XR implementation in higher education.

Further research should also investigate the technical and pedagogical challenges associated with XR adoption. Identifying these challenges could lead to detailed, actionable guidelines that institutions can use to optimize XR deployment strategies. Beyond health care education, exploring XR’s potential in fields such as engineering, humanities, and business would provide insights into its broader applicability. Moreover, examining how XR interacts with emerging technologies such as artificial intelligence, machine learning, and data analytics may reveal innovative ways to enhance teaching and learning experiences.

Another critical area for future research is the development of performance indicators to measure the success of XR adoption. These indicators should assess learning outcomes, user satisfaction, cost-effectiveness, and scalability, providing institutions with data-driven benchmarks to evaluate and refine their XR initiatives.

Finally, addressing the digital divide in XR adoption is crucial, particularly in developing regions. Investigating how educational institutions can ensure equitable access to XR technologies for students from varied socioeconomic backgrounds will help create inclusive and accessible learning environments. This research will be instrumental in bridging technological disparities and promoting digital equity in higher education.

### Conclusion

XR technologies have the potential to revolutionize health care education by providing immersive learning experiences that enhance practical skills and knowledge retention. This study highlights several key factors for the successful adoption of XR in medical education, including professional development, adequate infrastructure, robust technical support, positive social influence, and user-friendly design. Strategic investments in these areas are vital to overcoming initial barriers and aligning XR adoption with the Sustainable Development Goals of quality education and good health. By addressing these complex factors, educational institutions can create an environment conducive to the successful integration of XR technology, ultimately improving teaching practices and student learning outcomes in medical and nursing programs, particularly in Palestine.

The findings of this study emphasize that successful XR adoption in medical education requires more than just technological availability—it demands strong institutional policies, sustained funding mechanisms, and structured faculty development programs. Higher education institutions must move beyond pilot initiatives and develop long-term strategies for integrating XR into curricula, supported by clear guidelines, resource-sharing models, and institutional incentives. Additionally, regional collaborations among universities in developing contexts could facilitate knowledge exchange and infrastructure sharing, reducing the financial burden on individual institutions. Future research should further explore **scalable policy interventions** that enable sustainable XR adoption, particularly in resource-constrained environments where technology-enhanced learning can play a crucial role in addressing educational inequalities.

## Appendix

Multimedia Appendix 1 Interview protocol.

Multimedia Appendix 2 A sample of the inductive thematic analysis used in this study.

## Abbreviations

AR: augmented reality
MR: mixed reality
TAM: Technology Acceptance Model
UTAUT: Unified Theory of Acceptance and Use of Technology
VR: virtual reality
XR: extended reality
