# Supplementary material for: Factors Influencing Educators’ Perspectives on Accepting Extended Reality in Health Care Education: Qualitative Study
Source: JMIR Med Educ. 2025 May 1;11:e65042. doi: 10.2196/65042 (PMC12082055; doi:10.2196/65042)
Supplement: Multimedia Appendix 1 [file mededu_v11i1e65042_app1.docx]

***Q1: Background and demographic questions***

What faculty position do you currently have within the University?

What courses do you typically teach?

How many undergraduate/graduate students do you typically teach in a semester?

What kinds of technology do you use in the classroom, and how long have you used them?

**Practice and experience questions**

***Q2: What was the nature of the XR used in your teaching?***

***Prompts***

Describe the XR experience you used in teaching (presentation, headset type, length, etc.).

When did the XR experience take place?

Who was involved in the experience (what student group, module, class, etc.)?

Where did the XR experience occur (simulation lab, classroom, lecture theatre, etc.)?

How does XR skill assist your teaching?

***Q3: Why did you decide to use XR, and what was the rationale behind the decision?***

***Prompts***

What were you trying to achieve by using XR?

Why was I-XR chosen as the teaching method? What benefits does it have over others?

What were your thoughts before implementing the technology in teaching?

Did these thoughts change as you continued to use technology in teaching?

How did you measure/evaluate the experience?

What are the differences between practicing in a XR skill teaching environment and practicing with traditional skill learning methods?

***Q4: What barriers and facilitators existed when using XR?***

***Prompts***

What internal knowledge did you draw upon when you were teaching using XR?

Describe the nature of any external support from colleagues or faculty that assisted.

Were there any barriers (internal or external) that existed which made XR implementation challenging?

Did you encounter any difficulties when learning how to conduct VR skill teaching? Can you explain more?

***Q5: What are your thoughts about using XR in education?***

***Prompts***

What are your thoughts on ***XR*** now that you have used the technology in teaching?

What are your thoughts on the impact of using ***XR*** in teaching for both yourself and your students?

Based on your experience of ***XR***, what other disciplines, skills, or domains do you think it has the potential to be utilized in?

***Q6: Is there anything you would like to add to the interview content that was not previously stated?***
