# Supplementary material for: Factors Influencing Educators’ Perspectives on Accepting Extended Reality in Health Care Education: Qualitative Study
Source: JMIR Med Educ. 2025 May 1;11:e65042. doi: 10.2196/65042 (PMC12082055; doi:10.2196/65042)
Supplement: Multimedia Appendix 2 [file mededu_v11i1e65042_app2.docx]

| **Text Segments** | **Main Idea** | **Theme** | **Subthemes** |
| --- | --- | --- | --- |
| Participant (D2): During my teaching experience in the Department of Dentistry, I typically use various technology tools to engage my students in activities and to create real-life problems related to the field. Usually, I hesitant to use a new technology in my teaching practice because it needs a lot of time to learn about it and how to use it in teaching, but this attitude gradually be a positive attitude toward the new technology. Although it takes time initially especially to design activities by it [XR], it eventually saves effort and time. There are challenges, like extra time needed for first-time design and curriculum development, but with practice, it becomes easier. This philosophy drives my motivation, along with my students' engagement and interaction in course activities. | Experience in using ICT in teaching  Saving effort and time  Attitudes  Student engagement and interaction  Needs more time  Developing curriculum | Internal factors  Technology  Features  Challenges  Design factors | Prior experience with technology  Perceived usefulness  Easy to use  Design activities  Student support |
| **Participant D11:** It is a good tool to design activities to assess my students, it depends on the type of the assignments (I mean individual or team work assignments) …I created assignments that enable students to assess each other. It [XR] enhances the virtual learning environment…I asked my students to practice removing layers of tissues without any risk XR is an amazing technology medical education component, altering the teaching method. I will continue using XR if the University supports using it and provides more equipment for the system. I like to see a VR-based virtual lab to enhance practical applications and to have surgery simulation lab. It is important to keep faculty members updated about new technology in our field such as AI, VR… and to have technical support, as well incentives for faculty using XR would be beneficial. I recommend XR for all faculty, not just Medical education but also for humanities, social, and educational sciences. XR provides students a golden opportunity to learn independently, engage, collaborate, and practice in a virtual environment. | Strategies to use XR in medical education  Design assignments  Justification of continuance intention to use XR  Benefits for faculty members and students | Teaching strategies  Assessments  Continuance intention to use XR | Assessment  Activities development |
| **Participant D16**: Initially, I hesitated to use XR to visualize organs because I felt it needed more time, but later I managed to demonstrate organs to my students. I sought help from a friend to start designing activities. It was difficult initially, but I created and reused one activity. In one session, I asked students to explore a website presenting 3-D models of human organs (Mozaik) intending to learn from it. Initially challenging, the students later designed part of the assignment, which helped me learn XR for other classes. I received a certificate for projects completed with my students. I designed activities with problems for students to solve. | Hesitation to use XR  Policy encourages usage of XR  Social influence  Colleague support  Design elements/curriculum  Strategy to use  Student support  Familiarization time  Type of XR use  Reusing existing activities  Design activities  Problem-based learning assignments | Attitude  Institution policy  Colleague influence  Social support  Strategies to use XR  Challenges | Attitudes  Contextual factors  Social influence  Social support  Strategies to use XR |
|  |  |  |  |
